# Cardioprotective activity of alcoholic extract of *Tinospora cordifolia (Willd.) Miers* in calcium chloride-induced cardiac arrhythmia in rats

**DOI:** 10.1016/S1674-8301(11)60038-9

**Published:** 2011-07

**Authors:** Ashish Kumar Sharma, Kunal Kishore, Divya Sharma, B.P Srinivasan, Shyam Sunder Agarwal, Ashok Sharma, Santosh Kumar Singh, Samir Gaur, Vijay Singh Jatav

**Affiliations:** a Department of Pharmacology, Gyan Vihar School of Pharmacy, Suresh Gyan Vihar University, Jagatpura, Jaipur (Rajasthan)302025, India; b Delhi Institute of Pharmaceutical Sciences & Research, Department of Pharmacology, Pushpvihar, Sector-III. New Delhi 110017, India.

**Keywords:** *Tinospora cordifolia*, arrhythmia, cardioprotection.

## Abstract

The present study investigated the antiarrhythmic activity of alcoholic extract of *Tinospora cordifolia (T. cordifolia)* in CaCl_2_ induced arrhythmia. CaCl_2_ (25 mg/kg) was administered by intravenous infusion (iv) to produce arrhythmia in rats. The animals were then treated with *T. cordifolia* extract (150, 250, and 450 mg/kg) and verapamil (5 mg/kg,iv). Lead II electrocardiogram was monitored. Plasma calcium, sodium and potassium levels were measured. In CaCl_2_ induced arrhythmia, heart rate was decreased by 41.10%, *T. cordifolia* at 150, 300, and 450 mg/kg decreased the heart rate by 26.30%, 29.16%, and 38.29%, respectively, and verapamil reduced the heart rate by 9.70% compared to the normal group. The PQRST waves were normalized and atrial and ventricular fibrillation was controlled in rats treated with verapamil and *T. cordifolia*. CaCl_2_ increased calcium and sodium levels and decreased potassium levels in blood. *T. cordifolia* dose-dependently decreased calcium and sodium levels and increased potassium levels. Hence, *T. cordifolia* can be used in antiarrhythmic clinical settings and beneficial in atrial and ventricular fibrillation and flutter and may be indicated in ventricular tachyarrhythmia.

## INTRODUCTION

Cardiovascular disease is currently the most common disease globally, exerting a heavy personal and economic toll. Cardiac arrhythmia is one of the most common types of heart disease that is a main cause of mortality (approximately 17 million). Cardiac arrhythmia was the condition in which the heart's normal rhythm is disrupted. Cardiac arrhythmias are associated with abnormal initiation of a wave of cardiac excitation, abnormal propagation of a wave of cardiac excitation, or some combination of the two. Cardiac arrhythmias can manifest themselves in many different ways, and the mechanism of an arrhythmia was not clear yet. Arrhythmias also can be classified by the heart rate. The drugs used in the treatment for the cardiac arrhythmia are called antiarrhythmic drugs such as quinidine, amiodarone, propafenone, veramapril and lignocaine, which are some of the examples of antiarrhythmic drugs[1].

Common arrhythmias, particularly atrial fibrillation (AF) and ventricular tachycardia/fibrillation (VT/VF), are a major public health concern. Classic antiarrhythmic (AA) drugs for AF are of limited effectiveness, and pose the risk of life-threatening VT/VF. For VT/VF, implantable cardiac defibrillators appear to offer a unique, and yet unsatisfactory, solution. Very few AA drugs have been successful in the last few decades due to safety concerns or limited benefits in comparison to existing therapy. The Vaughan Williams classification (one drug for one molecular target) appears too restrictive in light of the current knowledge of molecular and cellular mechanisms. New AA drugs such as atrial-specific and/or multichannel blockers, upstream therapy and anti-remodeling drugs are emerging. We focus on the cellular mechanisms related to abnormal Na^+^ and Ca^2+^ handling in AF, heart failure, and inherited arrhythmias, and on novel strategies aimed at normalizing ionic homeostasis. Drugs that prevent excessive Na^+^ entry (ranolazine) and aberrant diastolic Ca^2+^ release *via* the ryanodine receptor RyR2 (rycals, dantrolene, and flecainide) exhibit very interesting antiarrhythmic properties. These drugs act by normalizing, rather than blocking, channel activity. Ranolazine preferentially blocks abnormal persistent (*vs* normal peak) Na^+^ currents, with minimal effects on normal channel function (cell excitability and conduction). A similar “normalization” concept also applies to RyR2 stabilizers, which only prevent aberrant opening and diastolic Ca^2+^ leakage in diseased tissues, with no effect on normal function during systole. The different mechanisms of action of AA drugs may increase the therapeutic options available for the safe treatment of arrhythmias in a wide variety of pathophysiological situations[2].

*Tinospora cordifolia* (*T.cordifolia*) belongs to the menispermaceae, popularly known as “Giloya”, which is a Hindu mythological term that refers to the heavenly elixir that has saved celestial beings from old age and kept them eternally young. *T. cordifolia* is widely used in veterinary folk medicine/ ayurvedic system of medicine as a tonic, vitalizer and as a remedy for diabetes[3] and metabolic disorders[4]. Scientific reports have described immuno-modulatory[5], antidiabetic[6], anti-inflammatory[7], hepatoprotective[8], anti-allergic[9], and antioxidant activities of *T. cordifolia*[10]. *T. cordifolia* also inhibited lipid peroxidation[11].

The findings suggest that the alcoholic extract of *T. cordifolia* possesses a dose dependent cardioprotection against ischemia-reperfusion induced myocardial injury and the cardioprotection may be due to its free radical scavenging activity or indirectly by enhancing the endogenous antioxidant levels or by protecting Mg^2+^ dependent Ca^2+^-ATPase enzyme or by antagonizing free radical mediated inhibition of sarcolemmal Na^+^-K^+^-ATPase activity or by Ca^2+^ channel blocking activity. Cardioprotective activity of alcoholic extract of *T. cordifolia* in ischemia-reperfusion induced myocardial infarction in rats has been reported[12]. Hence, the present study was designed to evaluate the cardioprotective activity of alcoholic extract of *T. cordifolia* (*Willd.*) Miers in calcium chloride induced cardiac arrhythmia in rats.

## MATERIALS AND METHODS

### Animals

The experimental protocol was approved by Institutional Animal Ethical Committee (IAEC-III, Gyan Vihar School of Pharmacy) and Committee for the Purpose of Control and Supervision of Experiments on Animals No. 1234/a/08/CPCSEA. Wister Albino rats of either sex weighing 150-250 g were procured from the Animal House, Gyan Vihar School of Pharmacy, Mahal, Jagatpura, Jaipur (Rajasthan) India. The animals were housed under standard laboratory conditions of (21±2)°C temperature, relative humidity of 55% and 12:12 h-light:dark cycles during the study. The animals were given standard rat pellet and tap water *ad libitum*.

### Plant material

The plant was collected from the Jagatpura Government nursery, Jaipur (Rajasthan) India and the plant had been authenticated by the Department of Botany University of Rajasthan, Jaipur (Rajasthan) India, voucher no. RUBL2085.

### Extract preparation

Freshly collected *T. Cordifolia* whole plant was dried under shade and the dried material was milled to obtain a coarse powder. The alcoholic extract of the powder was prepared by the process of continuous extraction (Soxhlation). The ethanolic extract was dried at constant weight such that 1 g equivalent to 4.57 g of crude drug was obtained. The dried extract was dissolved in sterile saline and used for further investigation[12],[13].

### Drugs and chemicals

Ketamine (Themis Medicare Ltd, Mumbai, India), calcium chloride (Ranbaxy Fine Chemicals Limited, Mumbai. India), absolute alcohol (Changshu Yangyuan Chemicals, Jilin China) and verapamil (Piramal Healthcare Limited, Mumbai. India) were used in the current study.

### Experimental protocol

Rats were randomly divided into eight groups, each consisting of six animals with different treatments (***Table-1***). Alcoholic extract of *T. Cordifolia* whole plant was prepared in the ethanol. The rats were anesthetized by ketamine (80 mg/kg i.p)[14]. Cardiac arrhythmias were induced by a single intravenous injection of 10% CaCl_2_ (50 mg/kg)[15]. The induced arrhythmias were then analyzed for magnitude of initial bradycardia, onset, incidence and duration of the induced fibrillations. After the induction of the arrhythmia, the animal was allowed to recover completely (15-20 min) and the test compound *T. cordifolia* dried ethanolic extract dissolved in saline was injected at different doses (150, 250, 450 mg/kg) intravenously[12]. The effect of the test compound on the basal heart rate was then examined and the percentage change in the heart rate was calculated. Seven minutes later, the arrhythmogenic dose of CaCl_2_ was re-administered and the effect of the treatment on the induced arrhythmia parameters was evaluated as percentage change in the measured parameters or as protection or non-protection against the induced fibrillation[14]. Group 3 were treated with verapamil at a dose of 5 mg/kg[16]. A lead II electrocardiogram was monitored throughout the study by using Cardiart108DG (BPL) with sensitivity 20 mm/mV at a paper speed of 25 mm/s. Heart rates were expressed as beats per min. Plasma levels of sodium and potassium levels were measured by specific electrodes and calcium by complexometric procedure[15].

### Statistical analysis

Values were expressed as mean±SEM. To analyze differences in variables before and after treatment paired Student's *t*-test was used. Comparison between different groups was done using one-way ANOVA followed by Tukey-Kramer multiple comparison test. *P* values < 0.05 were considered statistically significant. Statistical analysis was done using Sigma Stat 3.5 & Sigma Plot 10.0.

## RESULTS

### Effect of 10% and 5% calcium chloride intravenous injection in rats

**Fig. 1:**
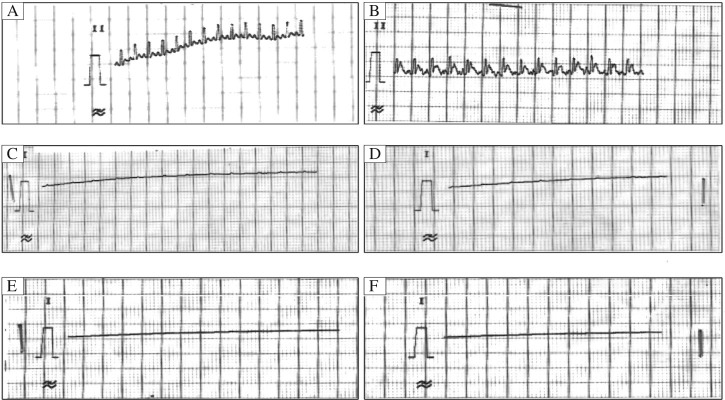
Representative electrocardiogram of Group II (10% CaCl_2_) at different time intervals. The ECGs were detected in Group II [Arrhythmia control group 1, 10% CaCl_2_ (50 mg/kg) (*n* = 6)]. with Dose 10% of CaCl_2_ (at different time intervals after intravenous administration). Arrhythmia, cardiodepression and mortality were observed at 20 min after 10% of CaCl_2_ administration. A: Normal reading (Control); B: Dose 10% of CaCl_2_ (at 0 min after administration) induced arrhythmic, C: Dose 10% of CaCl_2_ (at 5 min after administration) induced arrhythmic; D: Dose 10% of CaCl_2_ (at 10 min after administration) induced arrhythmic; E: Dose 10% of CaCl_2_ (at 15 min after administration) induced complete cardiodepression; F: Dose 10% of CaCl_2_ (at 20 min after administration) caused complete cardiodepression and mortality.

**Fig. 2:**
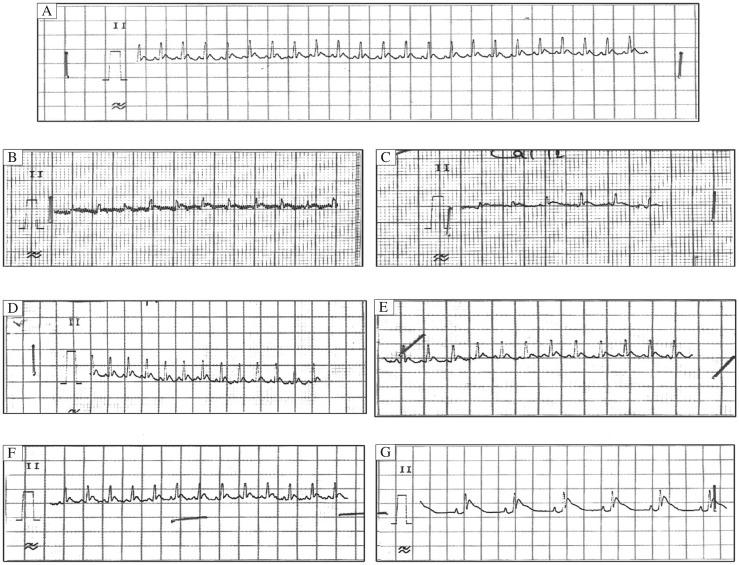
Representative electrocardiogram of different groups (Group I to Group VIII, *n* = 6) A: Normal reading (Control); B: Group III: Arrhythmia control [5% CaCl_2_ (25 mg/kg)]; C: Group IV CaCl_2_ (5%)-induced arrhythmia+alcohol (95%, 0.5ml, iv); D: Group V: CaCl_2_ (5%)-induced arrhythmia+verapamil (5 mg/kg, iv); E: Group VI CaCl_2_ (5%)-induced arrhythmia+*Tinospora cordifolia* dried ethanolic extract dissolved in saline.(150 mg/kg, iv); F: Group VII CaCl_2_ (5%)-induced arrhythmia+*Tinospora cordifolia* extract dissolved in saline (250 mg/kg, iv); G: Group VIII CaCl_2_ (5%)-induced arrhythmia+*Tinospora cordifolia* dried ethanolic extract dissolved in saline (450 mg/kg, iv) (*n* = 6)

**Fig. 3:**
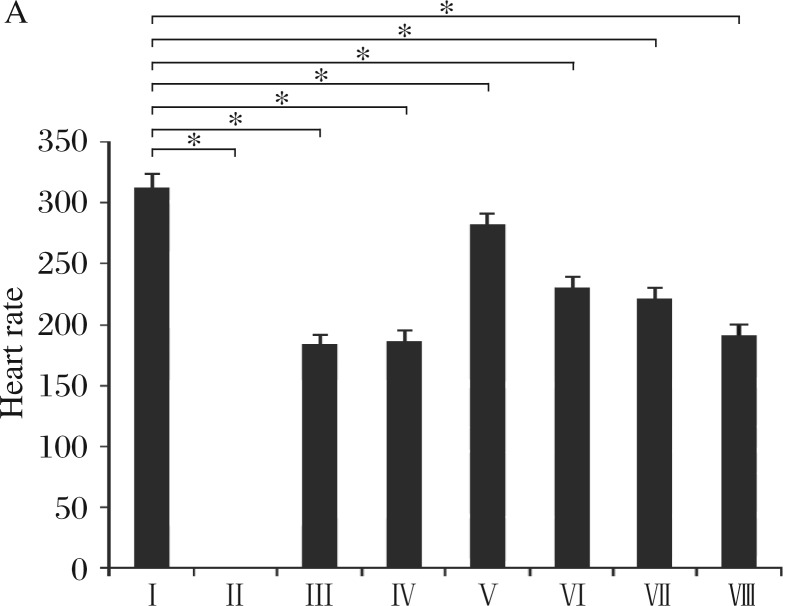
Comparison between the effects of *Tinospora cordifolia* extract and Verapamil on heart rate. *P < 0.05 for statistically significant *vs* control (Group I). (*n* = 6). Group I: Control; Group II: 10%CaCl_2_; Group III: 5%CaCl_2_; GroupIV: Alcohol 95%+5% CaCl_2_; Group V: Standard: CaCl_2_ (5%)+verapamil (5 mg/kg, iv); GroupVI: CaCl_2_(5%) + *Tinospora cordifolia* (150 mg/kg, iv); GroupVII: CaCl_2_(5%)+*Tinospora cordifolia* (250 mg/kg, iv); GroupVIII: CaCl_2_(5%)+*Tinospora cordifolia* (450 mg/kg, iv)

The experimental protocol is listed in ***Table 1***. In group II, intravenous injection of 10% CaCl_2_ (50 mg/kg)[15] caused decrease in the heart rate and showed alterations in the PQRST waves (***Fig. 1***). A lead II electrocardiogram showed changes in electrocardiogram including shortened QT interval, prolonged PR and QRS intervals, increased QRS voltage, T-wave flattening and widening, and notching of QRS. Atrial and ventricular fibrillation was demonstrated after 5 min of 10% CaCl_2_ (50 mg/kg) administration (***Fig. 1***). At 10 to 20 min, PQRST waves became more depressed. Ultimately, no wave occurred and mortality ensued. This showed that 10% CaCl_2_ (50 mg/kg) administration model of arrhythmia could not be managed. In group III, intravenous injection of 5% CaCl_2_ (50 mg/kg)[15] caused decrease in the heart rate and showed alterations in the PQRST waves (***Fig. 2B***). A lead II electrocardiogram showed changes in electrocardiogram including shortened QT interval, prolonged PR and QRS intervals, increased QRS voltage, T-wave flattening and widening, and notching of QRS. Atrial and ventricular fibrillation were demonstrated after 5 min of 10% CaCl_2_ (50 mg/kg) administration (ECG-1). At 10 to 20 min, the same pattern of depression of PQRST waves persisted. Ultimately, animals were recovered and there was no mortality. This showed that 5% CaCl_2_ (50 mg/kg) administration model produced desirable arrhythmia that could be managed. The percentage change in the heart rate of arrhythmic group was found to be decreased by 41.1% as compared to normal control (***Table 2***, ***Fig. 3***).

### The effects of 95% alcohol on 5% calcium chloride induced arrhythmias

In Group IV, intravenous injection of 0.5 mL of 95% alcohol showed no alterations in the PQRST waves (***Fig 2B***). A lead II electrocardiogram showed changes in electrocardiogram and included shortened QT interval, prolonged PR and QRS intervals, increased QRS voltage, T-wave flattening and widening, and notching of QRS. Atrial and ventricular fibrillation were demonstrated after 5 min of 0.5 mL of 95% alcohol administration (***Fig. 2C***). At 10 to 20 min, the same pattern of PQRST waves persisted. Ultimately, animals were recovered and there was no mortality. Furthermore, 0.5 mL of 95% alcohol administration as vehical produced no effect on arrhythmia in 5% CaCl_2_ (25 mg/kg) administration model, produced desirable arrhythmia and were not able to manage. The percentage change in the heart rate of arrhythmic group was found to be decreased by 40.5% as compared to normal control (***Table 2***; ****Fig. 3****).

### The effects of verapamil as standard drug on 5% calcium chloride induced arrhythmias

In the standard drug group (Group V), verapamil (5 mg/kg, iv)[17] was administered, which normalized the PQRST waves and hence atrial and ventricular fibrillation. In addition, the heart rate was increased as compared to the arrhythmic control group (***Fig. 2D***). The percentage changed in the heart rate was found to be decreased by 9.7% in the verapamil treated group as compared to normal control (***Table 2***; ***Fig. 3***).

**Table 1:** List of experimental groups and respective drug treatment along with the dose used (in Bracket)

Groups	Treatment (*(n* = 6)
Group I	Control (Rats on standard laboratory chow and tap water ad libitum)
Group II	Arrhythmia control group 1[10% CaCl_2_ (50 mg/kg)]
Group III	Arrhythmia control group 2 [5% CaCl_2_ (25 mg/kg)]
Group IV	CaCl_2_ (5%)-induced arrhythmia+alcohol (95%, 0.5 mL intravenously)
Group V	Standard: CaCl_2_ (5%)-induced arrhythmia+verapamil (5 mg/kg, iv)
Group VI	CaCl_2_ (5%)-induced anhythmia*+Tinospora cordifolia* dried ethanolic extract dissolved in saline (150 mg/kg, iv)
Group VII	CaCl_2_ (5%)-induced anhythxma*+Tinospora cordifolia* dried ethanolic extract dissolved in saline (250 mg/kg, iv)
Group VIII	CaCl_2_ (5%)-induced arrhythmia*+Tinospora* *cordifolia* dried ethanolic extract dissolved in saline (450 mg/kg, iv)

### The effects of extract of *T. Cordifolia* (150, 250 and 450 mg/kg)[12] on 5% calcium chloride induced arrhythmias

After calcium chloride induced arrhythmia, *T. cordifolia* alcoholic extract was administered intravenously at the different doses, which normalized the PQRST waves and hence atrial and ventricular fibrillation. In addition, the heart rate was increased as compared to the arrhythmic control group (***Fig. 2E***). In *T. Cordifolia* extract (150 mg/kg) treated group (Group VI), the percentage changed in the heart rate was found to be decreased by 26.3% as compared to normal control (***Table 2***, ***Fig. 3***). In *T. Cordifolia* extract (250 mg/kg) treated group (Group VII), the percentage changed in the heart rate was found to be decreased by 29.163% as compared to normal control (***Table 2***, ***Fig. 2F***, ***Fig. 3***). In *T. cordifolia* (450 mg/kg) treated group (Group VIII), the percentage changed in the heart rate was found to be decreased by 38.291% as compared to normal control (***Table 2***, ***Fig. 2G***, ***Fig. 3***).

### The effects of *T. cordifolia* and verapamil on the calcium, sodium and potassium levels

The time-course of blood calcium level was monitored after CaCl_2_ infusion. In arrhythmia control animals, plasma calcium level increased regularly, plasma sodium level tended to increase but the potassium levels slightly decreased. In alcohol treated arrhythmic group, there was no significant change in plasma levels of calcium, sodium and potassium as compared to arrhythmic Group II. In verapamil treated standard group calcium decreased, plasma sodium level tended to decrease and plasma potassium level to increase. The extract of *T. cordifolia* showed their dose-dependent effects in rats: blood calcium decreased, plasma sodium level tended to decrease and plasma potassium level increased as the dose of *T. cordifolia* was increased (***Table 3***).

**Table 2:** Heart rates of different groups.

Groups	Heart rates (beats/min (*n* = 6)
Group I	312.00±11.99
Group II	(mortality, depressed heart rate)
Group III	184.00±4.00*
Group IV	186.00±7.93*^a^
Group V	282.00±6.63*^a^
Group VI	230.00±6.32*^a^
Group VII	221.00±8.28*^a^
Group VIII	191.00±10.64*^a^

Expressed as mean±SEM values (n = 6). *P < 0.05 for statistically significant *vs* control. *P < 0.05 for statistically significant *vs* control arrhythmic (Group III).

## DISCUSSION

Arrhythmias produced by CaCl_2_ are severe and recalcitrant to manipulation, and rather high doses of antiarrhythmic drugs have to be given to produce a significant effect[18]. Moreover, the mechanism by which CaCl_2_ exerts its arrhythmogenic action is complex and not fully understood. It is due at least in part to an indirect action mediated by the autonomic nervous system. Initially, CaCl_2_ induces a cholinergic intervention[19]. The significance of a direct cardiac action is shown by the fact that, with increase of calcium concentration, severe arrhythmias, including ventricular fibrillation, occur also in the isolated heart[20],[21]. An increased calcium concentration induces a hyperpolarization of the resting potential in cells of the sinoatrial node[22] and a decrease in excitability of Purkinje cells due to a less negative value of the threshold potential. Moreover, repolarization of Purkinje cells is accelerated[23], as well as the repolarization of non-specialized myocardial fibers[24]. This difference in sensitivity leads to an asynchrony of recovery of excitability for different regions of the heart and could contribute to arrhythmia formation particularly by promoting re-entry. In the present study, intravenous injection of 10% CaCl_2_ given to the animals caused mortality (Group II), so 5% CaCl_2_ solution (25 mg/kg) that could induce arrhythmia without causing mortality was administered to the animal intravenously and their heart rates were monitored throughout the study by a lead II electrocardiogram. CaCl_2_ (5%)-induced arrhythmia+alcohol (95%, 0.5 mL, iv) was given in Group IV showed no significant effect of alcohol in CaCl_2_ (5%)-induced arrhythmia (***Fig. 2C***). CaCl_2_ (5%)-induced arrhythmia + verapamil (5 mg/kg. iv) showed normalization of PQRST waves and potent anti-arrhythmic effect of verapamil as compared to Group III (arrythmia control). The extract of *T. cordifolia* given intravenously at different doses (150, 250, and 450 mg/kg) (Group VI, Group VII, Group VIII, respectively) and the change in the PQRST waves was monitored. Different doses of *T. cordifolia* normalized the PQRST waves and hence atrial and ventricular fibrillation. In addition, the heart rate was increased as compared to the arrhythmic group (Group III). The effect of *T. cordifolia* was dose dependent; as the dose was increased the extract showed increased effect as reflected by progressive decrease in plasma calcium and sodium levels and increase in potassium levels at higher doses. In the standard drug group, verapamil (5 mg/kg iv)[12] was administered, which normalized the PQRST waves and hence atrial and ventricular fibrillation. In addition, the heart rate was increased. The comparison was done between *T. cordifolia* and verapamil in the arrhythmic and normal group and found that treatment with *T. Cordifolia* was as potent as verapamil treated group. High doses of *T. cordifolia* had dose-dependent antiarrhythmic action. The time-course of blood calcium level was monitored during CaCl_2_ infusion. In control arrhythmic animals, plasma calcium level was increased regularly, and plasma sodium level tended to increase but potassium levels were slightly decreased. The extract of *T. cordifolia* and verapamil showed dose-dependent effect in rats as blood calcium levels decreased, plasma sodium levels tended to decrease and plasma potassium levels increased as the dose of *T. cordifolia* increased. This indicated that *T. cordifolia* normalizes the arrythmiogenic calcium overload, decrease calcium-induced sodium levels and increased anti-arrhythmic potassium levels. In comparison to verapamil, *T. cordifolia* has additional effect on sodium and potassium levels *in vivo*.

**Table 3:** Ionic levels (mmol/L) in different groups after the treatment in calcium chloride induced arrhythmias

Groups	Calcium	Sodium	Potassium
Group I	1.01±0.03	36.80±0.40	90.00±0.40
Group II	5.85±0.60*	44.60±1.00*	88.60±1.20*
Group III	5.63±0.08*	42.73±0.14*	76.93±1.35*
Group IV	5.72±0.17*^a^	43.52±1.34*^a^	81.41±0.96*^a^
Group V	5.58±0.61*	37.90±1.00*	90.20±1.00*
Group VI	5.64±0.58*^a^	40.60±1.00*^a^	109.10±1.00*^a^
Group VII	4.02±1.83*^a^	39.86±1.74*^a^	113.46±2.34*^a^
Group VIII	3.49±3.67*^a^	30.52±2.33*^a^	123.67±4.03*^a^

Expressed as mean±SEM (*n* = 6). *P < 0.05 for statistically significant *vs* control. ^a^*P* < 0.05 for statistically significant *vs* control arrhythmic (Group III).

The extract of *T. cordifolia* showed potent antiarrhythmic activity by normalization in the PQRST waves as indicated by percentage of protection as compared with verapamil. Hence, *T. cordifolia* treatment may be used in antiarrhythmic clinical settings and beneficial for atrial and ventricular fibrillation and flutter. *T. cordifolia* decreases heart rate as shown by data more than verapamil, indicating that *T. cordifolia* may be indicated in ventricular tachyarrhythmias.
